# Analysis of Chemical Contaminants in Food

**DOI:** 10.3390/toxics8020027

**Published:** 2020-04-08

**Authors:** Claudio Medana

**Affiliations:** Molecular Biotechnology and Health Sciences Department, Università degli Studi di Torino, Via Pietro Giuria 5, 10125 Torino, Italy; claudio.medana@unito.it

Food chemical analysis is recognized as a unique tool for the characterization of nutritional value, quality and safety of foods and feeds. It is of growing importance to have an accurate knowledge of the global chemical composition of food and in particular of the chemical entities known as molecular (bio)markers. Quantitative determination of chemical markers is needed for risk assessment both in food and in environmental research after toxicological characterization of natural and synthetic chemicals. The potential adverse effects of chemical species, i.e., their hazard, can be classified as acute, subacute, subchronic and chronic. However, in all cases, the risk management does refine the toxicological evaluation by the chemical quantitation and the toxicokinetic assessment.

On these bases, we proposed this special issue, in order to have a view on emerging analytical methodologies to perform toxicology marker determination. 

The drive from the great development of methods in the industrial, pharmaceutical and environmental fields has extended the impact of chromatographic, spectrometric and spectroscopic techniques to the study of composition and contamination of foods. The interdisciplinary nature of analytical laboratories has allowed the extension of existing applications to the targeted and untargeted measurement of minor food components. These molecules are useful as toxicity biomarkers and in describing contamination in general.

This special issue contains a limited anthology of these kind of methods, but is highly representative of a broad worldwide overview, by collecting authors from ten different countries and four continents. Very different analytes are described, from volatile compounds to heavy metals and from highly polar substances to classical heterocyclic and organic aromatic contaminants. A large variety of analytical techniques is represented, including sample preparation and clean-up methodologies; the main current chromatographic-hyphenated spectroscopies together with mass spectrometry are more frequently reported. Finally, a differentiated variety of foods was the subject of the presented works: meat, fishery products, fruits and miscellaneous beverages are included in the studied matrices. Some applications of foods that require special care, such as infant formulae and human breast milk, are also presented.

Summarizing the highlights of this special issue:

- Okaru and Lachenmeier focused the attention on potential human carcinogenic compounds occurring in beverages, both newly formed by food processing, such as furfuryl alcohol, and naturally present, such as myrcene, a well-known plant metabolite [1]. NMR spectroscopy was employed for quantitative evaluation and obtained data were compared with a review of the literature on the topic.
- The work of Zeiner and Juranović Cindrić is centered on the determination of metal contaminants in Croatian wild fruits, such as lingonberries, blueberries and rosehips. Microwave digestion of samples and subsequent ICP-AES (Inductively coupled plasma–atomic emission spectroscopy) determination of metal species are discussed. Aluminum, nickel and lead levels were contextualized by reporting the results obtained for analyzed samples and wild fruit consumption as a function of acceptable intake [2].
- Fattore et al. applied complementary mass spectrometry-based methods (HPLC-MS/MS and HRGC-HRMS) to investigate polyhalogenated contaminants of Mediterranean marine fish and seafood of commercial interest at different trophic levels of the food chain. In particular, the authors searched for perfluorinated acids and brominated dioxins and furans as persistent organic pollutants, showing a low but noteworthy concentration of these molecules [3].
- Nuñez et al. proposed a useful spectral discrimination of isomeric food pollutants in order to distinguish compounds with high structural similarity. Isomeric compounds share elemental composition and mass value and are not distinguishable by first generation mass spectrometry, even if high resolving power instruments are used. By means of tandem mass spectrometry (MS/MS), the studied isomer pairs are clearly differentiated on the basis of the different fragmentation pathways of the corresponding ions formed in MS ionization [4].
- Raina-Fulton and Mohamad faced the problem of challenging food matrices as nutraceutical products. The necessity to overcome the presence of large number/concentrations of interferents leading to a severe matrix effect in HPLC-MS encouraged the authors to develop a complete analysis procedure. Applying these concepts to conazole fungicides as contaminants, a complete pathway was developed. At first the preanalytical procedure (pressurized extraction) was studied to optimize clean-up, then a HPLC-MS method was developed to identify conazole fungicide residues in Matcha tea [5].
- In their paper, Tran-Lam et al. applied a central composite design (CCD) to select the most valued operating condition to perform the extraction and gascromatographic-mass spectrometric determination of plasticizers in non-alcoholic beverages in Vietnam. The method was successfully applied to the determination of ten phthalate esters, obtaining satisfactory recoveries and limits of determination [6].
- The work of Lachenmeier et al. exploited analytical chemistry to monitor the relative concentration of four heat-induced coffee contaminants; acrylamide, furfuryl alcohol (FA), furan and 5-hydroxymethylfurfural (HMF). Different roasting modes were studied showing a direct/inverse relationship between roasting degree and analyte concentration depending on the molecule [7].
- The modulation of extraction parameters, the use of demulsifiers and the validation of the analytical performances were applied by Vichapong et al. to the analysis of polycyclic aromatic hydrocarbon residues (potential carcinogens formed by cooking) in grilled pork samples from Thailand [8].
- Vella and Attard described a multi-analyte and multi-technique study to characterize infant foods. Markers of toxicity may be neo-formed contaminants or derived from raw materials or processing and were quantified using various techniques. UV and ICP-AES spectroscopy applications were proposed and applied to evaluate both metal and HMF contaminants in infant foods and formulae sold in Malta [9].
- The paper of Smadi et al. reports the result of a study aimed to detect toxic residues in breast milk of Syrian refugee lactating mothers in Lebanon. The aim of the project was the potential identification of a relationship between antibiotic and pesticide residues and socio-demographic characteristics, dietary and smoking habits and medical history of participating lactating mothers. The results considered the breast milk collected from Syrian refugee lactating mothers as safe from chemical contamination [10].
- Finally, Dal Bello et al. reported the development of mass spectrometry methods (HPLC-MS and GC-MS) to detect illicit treatment of fishery products with hydrogen peroxide. Quantitation of residues of hydrogen peroxide was indirectly achieved by GC-MS monitoring of the reaction product between H_2_O_2_ and anisole to afford guaiacol. Biogenic amines, such as trimethylamine-N-oxide (TMAO), trimethylamine (TMA), dimethylamine (DMA), and cadaverine (CAD), were detected and measured by ion pair HPLC-MS. The developed analytical methods were validated and shown to be suitable to detect the illicit management of fishery products with hydrogen peroxide [11].

In summary, this collection of research articles provides a valuable selection of tools for food investigators to compare analytical methodologies and applications useful in the evaluation of toxicity markers. 
